# Transcriptional Activation of *Gstp1* by MEK/ERK Signaling Confers Chemo-Resistance to Cisplatin in Lung Cancer Stem Cells

**DOI:** 10.3389/fonc.2019.00476

**Published:** 2019-06-11

**Authors:** Jingyuan Li, Ting Ye, Yongli Liu, Liangsheng Kong, Zhiwei Sun, Doudou Liu, Jianyu Wang, H. Rosie Xing

**Affiliations:** ^1^Laboratory of Translational Cancer Stem Cell Research, Institute of Life Sciences, Chongqing Medical University, Chongqing, China; ^2^State Key Laboratory of Ultrasound Engineering in Medicine, Chongqing Medical University and the Ministry of Science and Technology, Chongqing, China

**Keywords:** *Gstp1*, cancer stem cell, chemotherapeutic resistance, MEK/ERK signaling pathway, lung adenocarcinoma

## Abstract

Lung cancer management remains a challenge due to its asymptomatic and late presentation when it is metastatic. The clinical response to the first-line platinum-based chemotherapy in patients with advanced lung cancer is disappointing due to the development of chemoresistance. Chemoresistance is a complex phenomenon. Mechanistic research using experimental models has yielded limited clinical results to help increase understanding for overcoming resistance. While the role of lung CSCs in conferring multidrug resistance has been postulated, experimental evidence remains associative and lacks in depth mechanistic inquisition. In the present study, using mouse and human lung adenocarcinoma cell lines and their respective paired CSC derivative cell lines that we generated, we identified cancer stem cell component of lung adenocarcinoma as the source that confers multidrug resistance phenotype. Mechanistically, *Gstp1* confers cisplatin resistance in mouse and human lung CSC models, both *in vitro* and *in vivo*. Further, transcriptional activation of *Gstp1* expression by MEK/ERK signaling underlies cisplatin resistance in lung CSC cells. Moreover, we show that *GSTP1* expression is a poor diagnostic and prognostic marker for human lung adenocarcinoma, thus is of high clinical relevance. Taken together, we have provided mechanistic understanding of the lung CSC in mediating chemoresistance.

## Highlights

- The cancer stem cell component of lung adenocarcinoma is the source that confers multidrug resistance phenotype.
- *Gstp1* expression is elevated in lung CSC cells which can be further increased upon treatment with a panel of chemotherapy drugs.
- *Gstp1* confers cisplatin resistance in mouse and human lung CSC models, both *in vitro* and *in vivo*.
- Providing the first experimental evidence that transcriptional activation of *Gstp1* expression by MEK/ERK signaling underlies cisplatin resistance in lung CSC cells.
- *GSTP1* expression is a poor diagnostic and prognostic marker for human lung adenocarcinoma thus is of high clinical relevance.

## Introduction

Lung cancer is the most common cause of cancer-related deaths in the world (1). The high mortality rate (51–99%) of lung adenocarcinoma is due to it being asymptomatic, it having late presentation when it is metastatic and becoming resistant to anti-cancer therapies (2). In spite of the development of new therapeutic strategies, the outcome of patients with metastatic lung cancer has barely improved over the past few decades, and the overall 5-year survival rate remains very low (10–15%) (3, 4). Lung adenocarcinoma is the most common histological type of lung cancer, comprising ~60% of non-small cell lung cancers (NSCLC) (5). Although platinum-based chemotherapy represents the standard first-line treatment for patients with advanced NSCLC, therapeutic outcome is disappointing due to the development of chemo-resistance, relapse, and distant metastases (6, 7). Mechanistic understanding of the involvement of commonly studied multidrug resistant genes using human lung adenocarcinoma cell lines has yielded limited clinical success in overcoming chemo-resistance thus far.

According to the CSCs theory, tumorigenesis, and cancer progression are due to a subset of phenotypically distinct cells characterized by unlimited self-renewal and enhanced clonogenic potential (8–10). Lung CSCs are shown to be associated with higher recurrence rates (11, 12). In agreement with this hypothesis, lung cancers that manifest stem cell signatures are associated with multidrug resistance (including cisplatin) and with disease relapse (12–14). However, in depth characterization and mechanistic investigation of multidrug resistance in lung CSCs were lacking, partially due to the lack of stable cellular models of lung CSC.

Glutathione S-transferases (GSTs) are phase II detoxifying enzymes involved in the maintenance of cell integrity, oxidative stress and protection against DNA damage by catalyzing the conjugation of glutathione to a wide variety of electrophilic substrates (15–17). *Gstp1*, a member of the GST family, is directly involved in the detoxification of cisplatin via the formation of cisplatin-glutathione adducts, which indicates that *Gstp1* may play a role in the acquisition of resistance to this platinum compound (18, 19). Even though a growing number of studies have demonstrated that *Gstp1* plays a key role in the development and maintenance of malignancy in several tumor types (20–22), mechanistic understanding of *Gstp1* in mediating chemoresistance in lung cancer is sketchy. Its role in mediating chemoresistance in CSCs is unknown.

The MAPK pathway, including MEK/ERK, JNK, and p38 kinase, plays a pivotal role in cell survival, proliferation and migration of tumor cells (23–25). While several studies reported activation of the MEK/ERK cascade in response to cisplatin treatment in several forms of cancer, the consequence of such activation on cell survival remains controversial (26–32). Few studies reported the activation of GST gene expression by MEK/ERK signaling in breast cancer (33–35). Up until the present study, regulation of *Gstp1* expression in lung CSCs has not been examined.

In the present study, we employed the lung CSCs derived from mouse parental Lewis lung carcinoma cell line (LLC-Parental) and human cancer cell lines H1299, which were named LLC-SD and H1299-SD, respectively. The stem cell properties of LLC-SD *in vitro* and *in vivo* had been characterized (36–38). Using the stable mouse and human lung CSC models that we generated and characterized, we clearly revealed lung CSCs as the cellular component that confer multidrug chemoresistance both *in vitro* and *in vivo*. We also demonstrate that the underlying mechanism of cisplatin chemo-resistance is the transcriptional activation of *Gstp1* by MEK/ERK signaling. We also show the clinical relevance of these findings and establish *GSTP1* as a poor diagnostic and prognostic marker, as well as a therapeutic target for human lung adenocarcinoma. Since the expression changes of *Gstp1* in response to chemo-therapeutic agents were specific to the lung CSCs, not the non-stem-like parental cells, *Gstp1* may also serve a lung CSC marker for evaluating the CSC content in clinical lung samples.

## Materials and Methods

### Cell Culture and Cell Lines

LLC-Parental and H1299-Parental cells were purchased from the Cell Bank of the Chinese Academy of Sciences (Shanghai, China) and cultured in DMEM high glucose supplemented (Hyclone) containing 10% FBS (Gibco). LLC-SD and H1299-SD cells were stem counterpart cells, which were maintained in DMEM/F12-based normal stem cell media (Hyclone), supplemented with 20 ng/ml EGF (BD), 20ng/ml FGF (BD) and 2% B27 (Gibco). All cell lines were cultured at 37°C, in a 5% CO_2_ atmosphere with 95% humidity.

### Reverse Transcription Quantitative Real-Time Polymerase Chain Reaction (RT-qPCR)

Total RNA was isolated using TRIZOL (Takara) according to the manufacturer's instructions. cDNA was reverse transcribed from 2 μg of total RNA. 39 cycles of PCR amplification were performed using 95°C for 30s, 95°C for 5s, and 60°C for 30s for each cycle. TBP was used as a loading control. The sequences of PCR primers are listed in Table 1.

**Table 1:** PCR primer sequence.

**Gene name**	**Forward primers**	**Reverse primers**
Mouse *Gstp1*	TATGTCACCCTCATCTACACCAACT	AGCAAGTTGTAATCGGCAAAGGAGA
Mouse *Abca2*	CCCGTCATGCAGTCGCTTT	CACTGGGTCGAACAAATTGCC
Mouse *Mrp1*	TGATGGCTCTGATCCACTCT	TCCACAGAAAGAATCCTAAGGCA
Mouse *Abcg2*	GAACTCCAGAGCCGTTAGGAC	CAGAATAGCATTAAGGCCAGGTT
Mouse *Mdr1*	CTGTTGGCGTATTTGGGATGT	CAGCATCAAGAGGGGAAGTAATG
Mouse *Mek1*	GTGCAGTCGGACATCTGGAG	CCACATGGCATCCAAACAGT
Mouse *Mek2*	ACATGGATGGTGGCTCACTG	CTGGTGCTTCTCTCGGAGGT
Mouse *Tbp*	GCGACCCTCACATCAAACT	CAGTGCCACATACCAACT
Human *GSTP1*	GAGGACCTCCGCTGCAAATAC	CTGGGACAGCAGGGTCTCAA
Human *TBP*	TATAATCCCAAGCGGTTTGC	CACAGCTCCCCACCATATTC

### Flow Cytometric Apoptosis Assay

At 24 h following transfection or inhibitor treatment, the indicated cells were treated with cisplatin at a final concentration of 10 μM. After 24 h of cisplatin treatment, the floating and attached cells were collected separately and then combined. 0.5–1 million cells of each single-cell suspension was stained with the Annexin V-fluorescein isothiocyanate (FITC) and propidium iodide (PI), and analyzed with a BD LSR. Unstained cells were included as negative controls for each FACS analysis. The data were analyzed using BD FACS software.

### Cck8 Assay and Drug Treatment

Cck8 colorimetric assay was used to detect the sensitivity of cells to anticancer drugs, cell viability and proliferation in response to drug treatments *in vitro*. They were also used to determine the concentration of the drug that inhibited cell growth by 50% (IC_50_) after 24 h of treatment. Cells were re-suspended in a final concentration of 2 × 10^3^ cells/well, seeded into 96 well plates and subjected to different concentrations of chemotherapeutic drugs after pre-incubation for 48 h. For drug combination treatment experiments, to investigate the reversal effects of cisplatin, different concentrations of cisplatin were added after pre-incubation with Ezatiostat or PD98059 for 48 h. After 24 h of incubation with cisplatin, Cck8 reagent (10 μl/well) was added and the plates were further incubated for 2 h. Subsequently, absorbance was determined at 450 nm by NanoQuant microplate reader (M200 PRO, Switzerland). The IC_50_ values (concentration required to inhibit the growth by 50%) were calculated from the survival curves using modified Bliss method. Resistance fold (Rf) was calculated by dividing the IC_50_ for the resistant cells with or without an inhibitor by that of the parental cells without an inhibitor. The concentrations of Ezatiostat or PD98059 used in this study were in the range of 5~150 μM.

### Western Blot

Western blot was performed according to a standard method. The total protein was extracted with RIPA buffer (Beyotime), according to the manufacturer's instructions. The concentration was determined by the BCA method. Twenty-five micrograms of protein was used to run on a 10% polyacrylamide (Beyotime) gel and transferred to a PVDF membrane underneath. The membrane was blocked in 5% non-fat milk (Bio-Rad). Primary antibodies and secondary antibodies (Proteintech) were used according to manufacturer's protocol. ECL method was used and the blot was developed under the gel electrophoresis imager (Bio-Rad). The following primary antibodies were used: anti–Gstp1 (Abcam), anti–Mek1 (upstate), anti–p-Mek1/2 (Cell Signaling Technology), and anti–GAPDH (Proteintech).

### siRNA Transient Interference Assay

To knockdown *Gstp1* or *Mek1/2* expression, LLC-Parental and LLC-SD cells were transfected with 5 nM control siRNA or siRNA against *Gstp1* (CCUCAUCUACACCAACUAUTT) or *Mek1/2* (AGUCGGACAUCUGGAGCAUTT) (GenePharma, China) for 72h using Lipofectamine 2000 transfect reagent (Invitrogen, USA).

### Lentivirus Packaging and Infection

Stable knockdown or overexpression of *Gstp1* in LLC-SD cells was achieved with infection of LLC-SD cells with either pLL3.7-shN.C.(mouse) or pLL3.7-shGstp1(mouse) and LV4-Vector(mouse) or LV4-OEGstp1(mouse), respectively, in the presence of 8 μg/ml polybrene (Genepharma, China) for 12 h, and the medium was refreshed. Seventy-two hours after the infection, the efficiency of infection was measured under a fluorescent microscope and by RT-qPCR.

### Clinical Samples

We collected 43 tumor samples from The Affiliated Hospital of Southwest Medical University after informed consent approved by Southwest Medical University of Institutional Review Boards. Specimens were prepared from paraffin embedded (FFPE) tissue. Genomic Total RNA from FFPE tissues was extracted using the Tiangen RNAprep Pure FFPE Kit (Tiangen, China). RNA yield was evaluated using a Nanodrop 2000 spectrometer (NanoDrop Technologies Inc., DE) and Qubit 3.0 Fluorometer (Thermo Fisher Scientific, MA). All of the formalin fixed paraffin embedded was obtained from the department of pathology in the affiliated hospital of southwest medical university, which was used to identify the cancer progress as the stage of lung cancer and received the approval of the institutional ethics committee of southwest medical university medical center. Moreover, this study obtained written informed consent from the participants.

### Cisplatin Resistance Assay

Single cell suspension (1 × 10^3^) mixed with 50 μL Matrigel Matrix (Corning) at a 1:1 ratio was injected subcutaneously into both insides of the hind legs of each 6–8 weeks old female BALB/c nude mice (Beijing Huafukang bioscience company, Beijing, China). Tumor growth was measured every 2 days, and tumor volume was calculated as V = (length × width^2^)/2. Treatment with cisplatin started when the tumor volume of the subcutaneous xenograft tumors reached about 100 mm^3^. 16 mg/kg cisplatin was administrated every other day intraperitoneally (qilu-pharmaceutical). After 9 days of treatment, when mouse body weights were decreased by 20%, mice were killed in a humane manner. Experimental mice were raised in an accredited specific pathogen-free Animal Facility at Chongqing medical University. All protocols were approved by the Institutional Animal Care and Use Committee of Chongqing medical University and conducted with humane animal care.

### Bioinformatics Analyses

The lung cancer dataset, which included 535 lung adenocarcinoma tissues and 59 normal lung tissues, was obtained from The Cancer Genome Atlas project (TCGA, https://portal.gdc.cancer.gov/). Student's *t*-test was then used to compare *GSTP1* expression levels between lung cancer and normal lung tissues. Gene set enrichment analysis (GSEA) was performed to identify pathways associated with *GSTP1* mRNA expression levels in the TCGA lung cancer dataset. GSEA software was obtained from the Broad Institute (http://www.broad.mit.edu/gsea).The expression levels of *GSTP1* genes in the selected cancers were analyzed using Oncomine. For this, we compared clinical specimens of cancer vs. normal patient datasets. In order to reduce our false discovery rate, we selected *p* < 0.01 and gene rank, with the top 10% as a threshold. We analyzed the results for their *p*-values, fold change, and cancer subtype. In many instances we found several significant correlations in different tumor types, but we showed only lung adenocarcinoma except for sub-forms. TCGA survival analysis used the website http://www.oncolnc.org/. Additionally, Kaplan-Meier Plotter database was used to draw the overall survival (OS), progression-free survival (FS) and post-progression survival (PPS) curves.

### Statistical Analysis

Data were analyzed by one-way analysis of variance and Student's independent *t*-test using GraphPad Prism software and was presented as mean ± SD or SEM indicated in figure legends. Differences were considered statistically significant when *P* < 0.05.

## Results

### The Multi-drug Resistant Phenotypes of Lung Cancer Stem Cells Are Manifested by Their Resistance to a Wide Range of Structurally and Functionally Unrelated Chemotherapy Drugs

**Figure 1:**
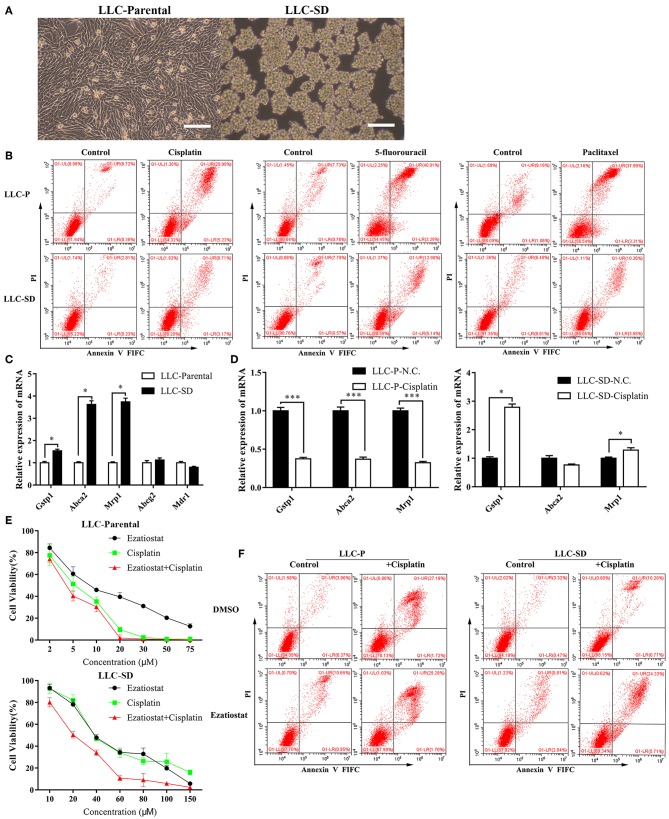
The multi-drug resistant phenotype of Lung cancer stem cells is manifested by their resistance to a wide range of structurally and functionally unrelated drugs. **(A)** Morphological differences between the spindle attached LLC-Parental cell line and its derivative CSC spheroid forming floating LLC-SD cell line, bar = 120 μm. **(B)** Annexin V-FITC/PI staining of apoptotic LLC-Parental and LLC-SD cells under treatment of cisplatin(10 μM), 5-fluorouracil(10 μM) and paclitaxel(10 μM) for 24 h. **(C)** The expression of resistance-related genes (*Gstp1, Abca2, Mrp1, Abcg2*, and *Mdr1*) in LLC-SD cell lines and LLC-Parental by RT-qPCR, the tata-Box binding protein gene (*Tbp*) was used as a reference gene. **(D)** The expression of resistance-related genes (*Gstp1, Abca2*, and *Mrp1*) after cisplatin treatment in LLC-SD cell lines by RT-qPCR, the tata-Box binding protein gene (*Tbp*) was reference gene. **(E)** Single-cell suspensions LLC-Parental and LLC-SD cell lines were seeded at 2,000/well in sphere medium and treated with increasing concentrations of cisplatin or *Gstp1* inhibitor Ezatiostat alone or in combination. After 24 h of treatment, Cell growth curves (%) was derived and compared to the DMSO control. **(F)** Annexin V-FITC/PI staining of apoptotic LLC-Parental and LLC-SD cells under treatment of cisplatin(10 μM) and/or Ezatiostat(5 μM) for 24 h. Results are statistically significant if ^*^p < 0.05, ^***^p < 0.001(Student's *t*-test).

The lung CSCs LLC-SD cells were derived from the LLC-Parental cell line and demonstrated high capability of self-renewal and enhanced tumorigenicity as we characterized and reported (36–38). LLC-SD cells exhibited spheroid growth *in vitro* and can be stably maintained in culture (Figure 1A). The multi-drug resistant phenotype of LLC-SD was manifested by their resistance to a wide range of structurally and functionally unrelated chemotherapy drugs. LLC-SD cells exhibited more than 3-folds more resistance to cisplatin, paclitaxel and 5-fluorouracil compared with that in LLC-Parental cells (Figure 1B). LLC-SD cells were more resistant to undergo apoptosis upon all drug treatments than the paired LLC-Parental cells (Figure 1B). LLC-SD exhibited 12.8-, 30.0-, and 50.6-fold resistance to cisplatin, paclitaxel and 5-fluorouracil, respectively, as determined by IC_50_ (Table 2). To discern the potential genes that mediate multidrug resistance in the LLC-SD cells, we examined the expression of known multidrug resistance genes, including *Gstp1, Abca2, Mrp1, Abcg2* and *Mdr1*, by RT-qPCR. *Gstp1, Abca2, Mrp1* expression was robustly upregulated in LLC-SD, compared with that in LLC-Parental (Figure 1C).

**Table 2:** Chemosensitivity of LLC-Parental/LLC-SD cells to different chemotherapy drugs.

**Drug**	**IC**_**50**_		**LLC-SD/LLC-Parental resistance ratio**
	**LLC-Parental**	**LLC-SD**	
Cisplatin	4.0 μM	51.0 μM	12.8
Paclitaxel	2.7 μM	80.9 μM	30.0
5-FU	3.1 μM	156.8 μM	50.6

Cisplatin is one of a number of platinum (Pt)-containing compounds used for the treatment of a variety of malignancies, including ovarian and testicular cancer, and remains the first-line of chemotherapy for patients with advanced NSCLC (39). Hence, we further determined the expression of *Gstp1, Abca2, Mrp1* before and after cisplatin treatment in LLC-SD and LLC-Parental cells. While the expression of all three genes in LLC-Parental cells was inhibited upon cisplatin treatment, *Gstp1* and *Mrp1* expression was elevated in LLC-SD cells after cisplatin treatment (Figure 1D) and the most pronounced increase was seen in *Gstp1* (Supplementary Figure 3). Thereafter, we chose to focus on the *Gstp1* gene and cisplatin for mechanistic investigation as GSTs are known to form adducts with platinum compound (18). Treatment of LLC-SD cells with an *Gstp1* inhibitor, Ezatiostat, reversed the resistance to cisplatin at all doses evaluated, while Ezatiostat had less of an effect on LLC-Parental cells determined by Cck-8 assay (Figure 1E). Flow cytometric analysis confirmed these findings (Figure 1F). Taken together, these observations demonstrate that it is the CSC component of the LLC-Parental cells, i.e., the LLC-SD cells, that confer resistance to cisplatin, which appears to be mediated by *Gstp1*.

### Blocking *Gstp1* Reversed Cisplatin Resistance Both *in vitro* and *in vivo*

**Figure 2:**
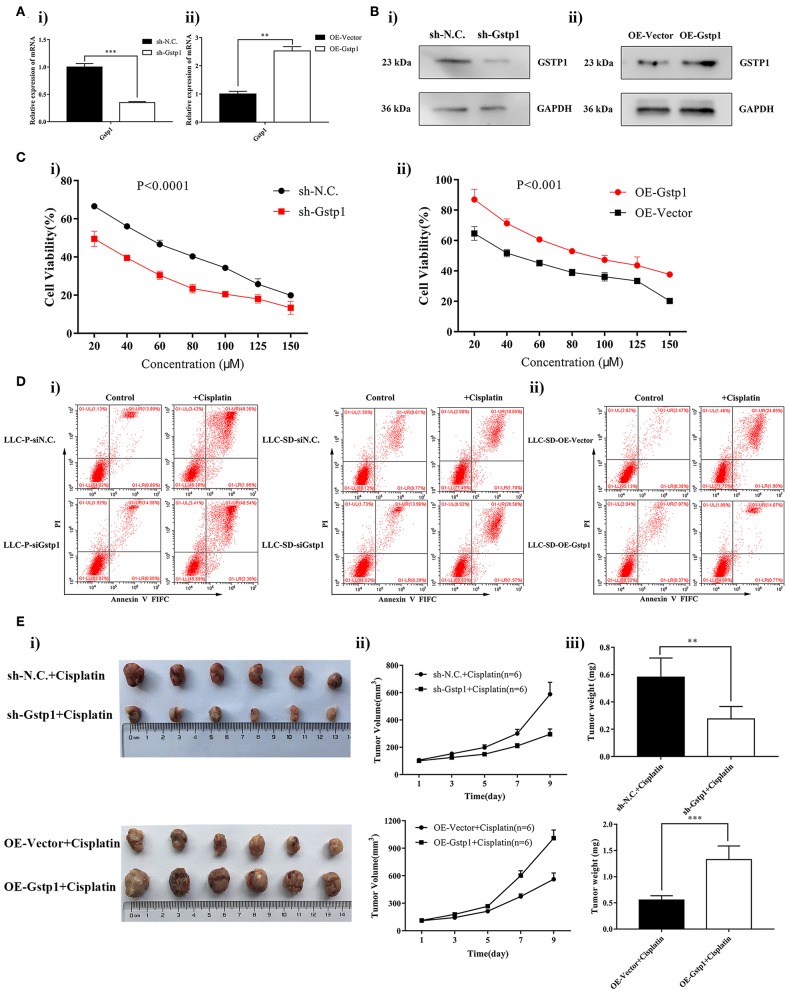
*Gstp1* regulates cisplatin sensitivity in LLC-SD cells. **(Ai,ii)** Expression of *Gstp1* in LLC-SD cells infected with control shRNA (shN.C.), shRNA to *Gstp1* (shGstp1), control Vector (Vector), and overexpression of *Gstp1* (OEGstp1) by RT-qPCR, with tata-Box binding protein gene (*Tbp*) as the reference gene, ^**^p < 0.01, ^***^p < 0.001. **(Bi,ii)** Expression of Gstp1 in shN.C., shGstp1, Vector, and OEGstp1 Group by western blotting, with GAPDH as the reference protein. **(Ci,ii)** Single-cell suspensions of shN.C. and shGstp1/Vector and OEGstp1 groups were seeded at 2,000/well in sphere medium and treated with increasing concentrations of cisplatin. Growth curves (%) was derived and compared to the DMSO control. **(Di,ii)** Annexin V-FITC/PI staining of apoptotic siN.C. and siGstp1/Vector and OEGstp1 groups under treatment of cisplatin(10 μM) for 24 h. **(E)** *Gstp1* expression regulates LLC-SD tumor sensitivity to cisplatin *in vivo*. Subcutaneous tumors harvested after cisplatin treatment in BALB/c nude mice on Day 9. **(i)** tumor volume; **(ii)** tumor growth curve; **(iii)** tumor weigh, ^**^p < 0.01, ^***^p < 0.001.

To confirm the involvement of *Gstp1* in mediating chemoresistance of lung CSCs, we deregulated Gstp1 expression by generating LLC-SD cell lines: (i) in which *Gstp1* was constitutively inhibited by lentiviral-mediated *Gstp1* shRNA (shGstp1) silencing, and (ii) in which Gstp1 was up-regulated by lentiviral-mediated *Gstp1* (OEGstp1) over-expression (Figures 2A,B). Chemosensitivity of LLC-SD to cisplatin varied with changes in *Gstp1* expression. Inhibition of *Gstp1* by shGstp1 resulted in a left-shifted cell survival curve indicating sensitization (Figure 2C, left). In contrast, *Gstp1* overexpression led to enhanced resistance to cisplatin, as evident by the right-shifted cell survival curve (Figure 2C, right). The Cck-8 assay results were confirmed by the apoptosis assay (Figure 2D).

To determine whether *Gstp1* mediates cisplatin resistance *in vivo*, four cell lines that differ in *Gstp1* expression were injected subcutaneously into the nude mice (**Methods**, 1000 cells/side). After 11 days of injection, when subcutaneous xenograft tumors growth reached ~100 mm^3^, cisplatin treatment was administrated (**Methods**). The experiment was terminated on Day 9 since the start of the treatment after 4 treatments wereadministered. Consistent with our *in vitro* finding, *Gstp1* regulates LLC-SD tumor sensitivity to cisplatin, measured by visual examination of tumor size (Figure 2Ei), tumor growth rate (Figure 2Eii) and tumor weight (Figure 2Eiii).

Taken together, *Gstp1* regulates LLC-SD sensitivity to cisplatin both *in vitro* and *in vivo* (Figures 1, 2).

### MEK/ERK Signaling Pathway Mediates Cisplatin Resistance in LLC-SD Cells

**Figure 3:**
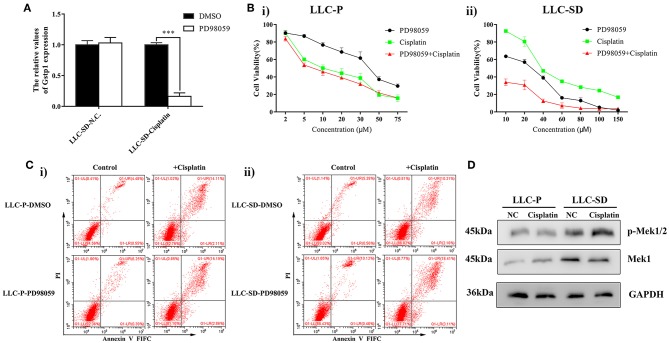
MEK/ERK inhibitor treatment sensitize LLC-SD to cisplatin. **(A)** Expression of Gstp1 in LLC-SD cells under treatment of cisplatin or PD98059 alone, or in combination by RT-qPCR, with tata-Box binding protein gene (*Tbp*) as the reference gene, ^***^p < 0.001. **(B)** Single-cell suspensions LLC-Parental and LLC-SD cell lines were seeded at 2,000/well in sphere medium and treated with increasing concentrations of cisplatin or PD98059 alone or in simultaneous combination. After 24 h of treatment, growth curves (%) were constructed and compared. **(i)** LLC- Parental; **(ii)** LLC-SD. **(C)** Annexin V-FITC/PI staining of apoptotic LLC-Parental and LLC-SD cells under treatment of cisplatin (10 μM) and/or PD98059 (5 μM) for 24 h. Results are statistically significant if ^***^p < 0.001(Student's *t*-test). **(i)** LLC-Parental; **(ii)** LLC-SD. **(D)** Expression of p-Mek1/2 and Mek1 in LLC-Parental or LLC-SD cell line after treatment with cisplatin by western blotting, with GAPDH as the reference protein.

It is reported that activation of the mitogen activated protein kinase (MEK/ERK) pathway may mediate resistance to cisplatin in ovarian cancer cells by regulating GST-π expression (40). Gene set enrichment analysis (GSEA) was performed to evaluate signaling pathways that were associated with *Gstp1* expression in the TCGA lung cancer samples. The results revealed that *Gstp1* expression was positively correlated with the MEK/ERK signaling pathway (Supplementary Figure 1A), which implied that *Gstp1* may be affected by MEK/ERK signaling pathway in lung cancer. To investigate the relationship between MEK/ERK signaling pathway and *Gstp1*, we first evaluated whether *Gstp1* expression is subjected to MEK/ERK signaling pathway regulation. Treatment of LLC-SD cells with MEK/ERK inhibitor PD98059, at the same time of receiving cisplatin treatment, prevented the up-regulation of *Gstp1* expression (Figure 3A). In LLC-SD cells that exhibit resistance to cisplatin both *in vitro* and *in vivo* (Figures 1, 2), LLC-SD proliferative activity was sensitive to the inhibition of the MEK/ERK signaling pathway by PD98059 (Figure 3Bii). Further, PD98059 treatment significantly reduced LLC-SD resistance to cisplatin (Figure 3Bii). In contrast, PD98059 treatment did not alter cisplatin sensitivity in LLC-P cells (Figure 3Bi) that were sensitive to cisplatin's growth inhibition (Figure 1B). In agreement with the Cck8 analyses, PD98059 augmented cisplatin-induced apoptosis in LLC-SD cells (Figure 3Cii), and it restored the ability of cisplatin to induce apoptosis in LLC-SD to a level that is comparable to cisplatin-induced apoptosis in LLC-P cells (Figure 3Ci). Based on this set of observations, we hypothesized that cisplatin-induced increase in *Gstp1* expression (Figure 1D), that was subjected to PD98059 inhibition (Figure 3A), might be a result of cisplatin-induced activation of the MEK/ERK signaling pathway leading to transcriptional activation of *Gstp1* expression. Consistent with this hypothesis, enhancement of Mek1 phosphorylation was only seen in cisplatin-resistant LLC-SD cells and not in the LLC-Parental cells that were sensitive to cisplatin (Figure 3D).

**Figure 4:**
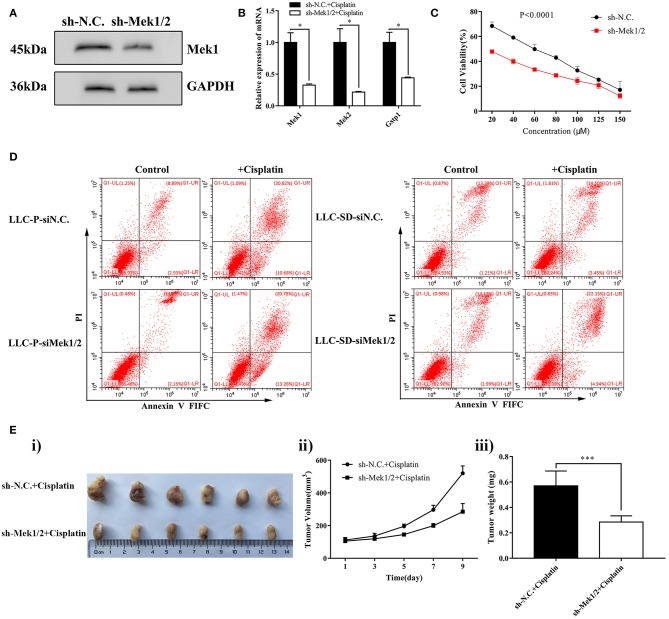
Stable inhibition of MEK/ERK signaling via shMek1/2 restored chemosensitivity to cisplatin in LLC-SD cells. **(A)** Expression of Gstp1 in LLC-SD-shN.C. and shMek1/2cells by western blotting, with GAPDH as a reference. **(B)** Expression of *Mek1, Mek2*, and *Gstp1* in shN.C. and shMek1/2 groups after treatment with cisplatin by RT-qPCR, with tata-Box binding protein gene (*Tbp*) as the reference gene, ^*^p < 0.05. **(C)** Single-cell suspensions of LLC-SD-shN.C. and LLC-SD-shMek1/2 cells were seeded at 2,000/well in sphere medium and treated with increasing concentrations of cisplatin. After 24 h of treatment, growth curves (%) were constructed and compared. **(D)** Annexin V-FITC/PI staining of apoptotic LLC-SD-siN.C. and siMek1/2 cells under treatment of cisplatin (10 μM) for 24 h. Results are statistically significant if ^*^p < 0.05, ^***^p < 0.001(Student's *t*-test). **(E)** Mek1/2 silencing restored LLC-SD tumor sensitivity to cisplatin *in vivo*. Subcutaneous tumors harvested after cisplatin treatment in BALB/c nude mice. **(i)** tumor volume; **(ii)** tumor growth curve; **(iii)** tumor weigh, ^***^p < 0.001.

To further investigate the role of the MEK/ERK signaling pathway in mediating cisplatin resistance in LLC-SD cells, we generated LLC-SD derivative cell lines in which Mek1/2 expression was stably inhibited by RNA interference (shMek1/2) (Figure 4A). Mek1/2 silencing prevented cisplatin-induced increase in Gspt1 expression in LLC-SD cells (Figure 4B). Consequently, cisplatin resistance was reduced, as determined by the Cck8 assay (Figure 4C) and apoptosis assay (Figure 4D) *in vitro* and by tumor growth and tumor weight *in vivo* (Figures 4Ei–iii), upon inactivation of the MEK/ERK pathway by Mek1/2-knockdown. This set of observations are consistent with PD98059 treatment, confirming the role of MEK/ERK signaling in mediating resistance to cisplatin in LLC-SD lung CSC cells.

### *Gstp1* Confers MEK/ERK Mediation of Cisplatin Chemoresistance in LLC-SD Cells

**Figure 5:**
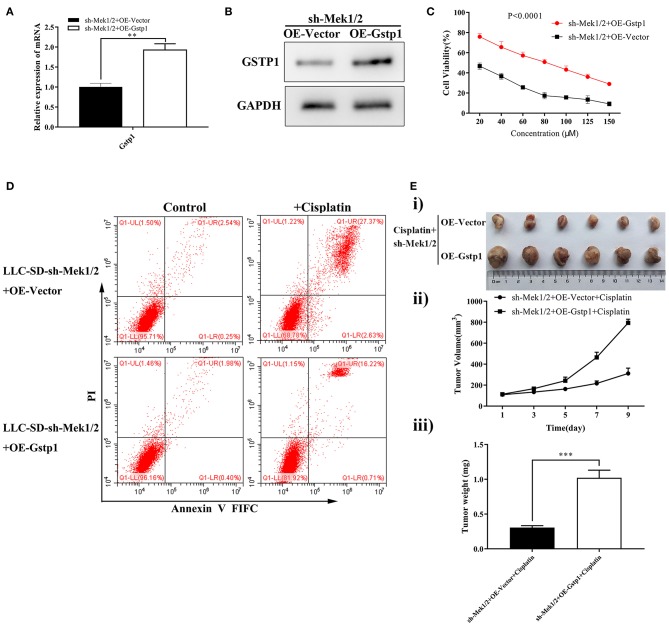
Overexpression of *Gstp1* in LLC-SD-shMek1/2 cells restored resistance to cisplatin. **(A)** Expression of *Gstp1* in LLC-SD-shMek1/2+Vector and LLC-SD-shMek1/2+OEGstp1 cells by RT-qPCR, with tata-Box binding protein gene (*Tbp*) as the reference gene. **(B)** Expression of Gstp1 in LLC-SD-shMek1/2+Vector and LLC-SD-shMek1/2+OEGstp1 cells by western blotting, with GAPDH as the reference protein. **(C)** Single-cell suspensions of LLC-SD-shMek1/2+Vector and LLC-SD-shMek1/2+OEGstp1 cells were seeded at 2,000/well in sphere medium and treated with increasing concentrations of cisplatin. After 24 h of treatment growth curves (%) were constructed and compared. **(D)** Annexin V-FITC/PI staining of apoptotic LLC-SD-shMek1/2+Vector and LLC-SD- shMek1/2+OEGstp1 cells under treatment of cisplatin (10 μM) for 24 h. **(E)** Overexpression of *Gstp1* in LLC-SD-shMek1/2 cells restored resistance to cisplatin *in vivo*. Subcutaneous tumors harvested after cisplatin treatment in BALB/c nude mice. **(i)** tumor volume; **(ii)** tumor growth curve; **(iii)** tumor weigh, ^**^p < 0.01, ^***^p < 0.001.

To order the molecular relationship between activation of MEK/ERK signaling and transcriptional activation of *Gstp1* in mediating cisplatin chemoresistance, *Gstp1* expression was restored in *Mek1/2* inhibited LLC-SD-shMek1/2 cells by lentiviral-mediated overexpression of *Gstp1* (Figures 5A,B). Restoration of *Gstp1* expression in LLC-SD-shMek1/2 cells reversed the cisplatin sensitive phenotype seen in LLC-SD-shMek1/2 cells to cisplatin resistance seen in shMek1/2+OEGstp1 cells, measured by Cck-8 proliferation assay (Figure 5C) and the apoptosis assay (Figure 5D) *in vitro*, and the tumor transplantation assay *in vivo* (Figure 5E). These results indicate that *Gstp1* acts downstream of MEK/ERK signaling pathway. Further, MEK/ERK signaling mediates cisplatin resistance in lung CSC LLC-SD cells by transcriptional activation of *Gstp1*.

### High *Gstp1* mRNA Levels Are Associated With Clinical Accelerated Disease Progression and Poorer Survival in Lung Adenocarcinoma

**Figure 6:**
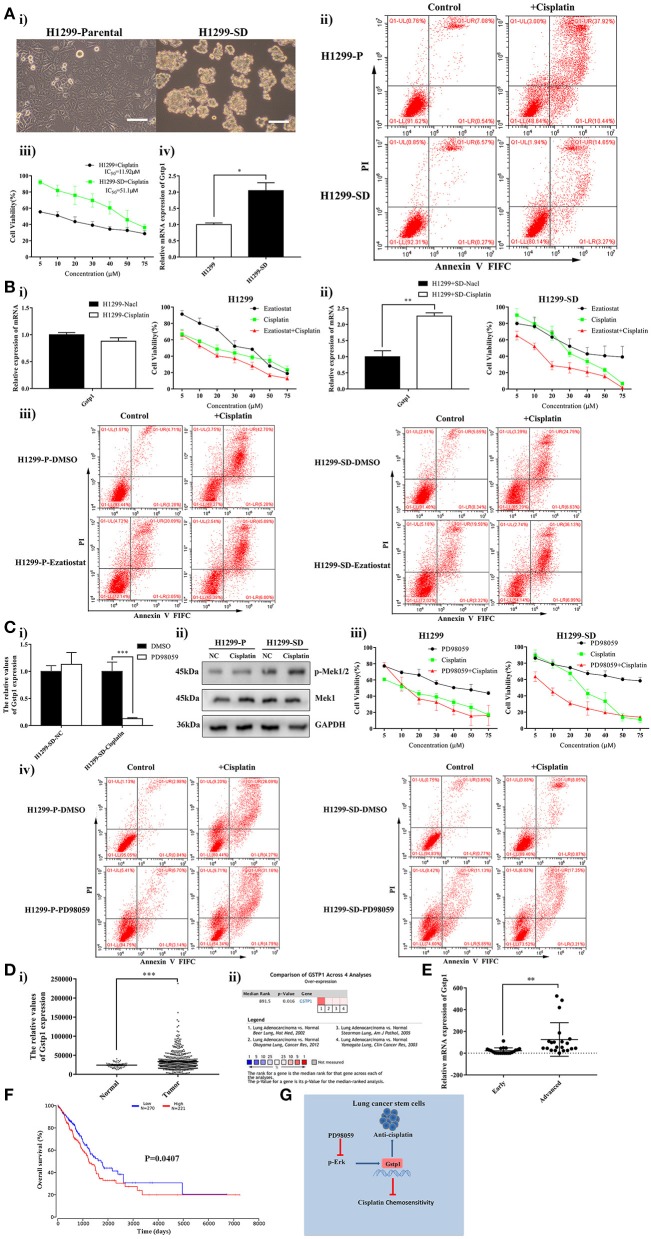
High *GSTP1* mRNA levels are linked with disease progression and poorer survival in lung adenocarcinoma. **(Ai)** Morphological differences between the spindle attached human lung adenocarcinoma H1299-Parental cell line and its derivative CSC spheroid forming floating H1299-SD cell line, bar = 120 μm. **(ii)** Annexin V-FITC/PI staining of apoptotic H1299-Parental and H1299-SD cells under treatment of cisplatin (15 μM) for 24 h. **(iii)** The IC_50_ assay of H1299-parental and H1299-SD. **(iv)** Expression of *GSTP1* in H1299-Parental and H1299-SD cell lines by RT-qPCR. **(Bi)** Expression of *GSTP1* after cisplatin treatment in H1299-Parental and H1299-SD cell lines, with tata-Box binding protein gene (*TBP*) as the reference gene, ^*^p < 0.05. **(ii)** Single-cell suspensions H1299-Parental and H1299-SD cell lines were seeded at 2,000/well in sphere medium and treated with increasing concentrations of cisplatin or Ezatiostat alone or in simultaneous combination. After 24 h of treatment growth curves (%) were constructed and compared. **(iii)** Annexin V-FITC/PI staining of apoptotic H1299-Parental and H1299-SD cells under treatment of cisplatin (15 μM) and/or *GSTP1* inhibitor Ezatiostat (10 μM) for 24 h. **(Ci)** Expression of *GSTP1* in H1299-SD cells under treatment of cisplatin or PD98059 alone or in simultaneous combination by RT-qPCR, with tata-Box binding protein gene (*TBP*) as the reference gene, ^***^p < 0.001. **(ii)** Expression of p-Mek1/2 and Mek1 in H1299-parental or H1299-SD cell line after treatment with cisplatin by western blotting, with GAPDH as the reference protein. **(iii)** Single-cell suspensions H1299-Parental and H1299-SD cell lines were seeded at 2,000/well in sphere medium and treated with increasing concentrations of cisplatin or PD98059 alone or in combination. After 24 h of treatment, the growth curves (%) were constructed and compared. **(iv)** Annexin V-FITC/PI staining of apoptotic H1299-Parental and H1299-SD cells under treatment of cisplatin (15 μM) and/or PD98059 (10 μM) for 24 h. **(Di)** *GSTP1* expression level in normal lung tissues and LUAD tissues in the mRNA sequencing dataset of LUAD from the TCGA (Normal, *n* = 59; Lung adenocarcinoma, *n* = 535). **(ii)** Meta-analysis of the 4 datasets from 4 studies (Beer Lung, Stearman Lung, Hou Lung, and Okayama Lung) on *GSTP1* mRNA levels in LUAD vs. normal lung tissue searched by Oncomine database. **(E)** A total of 43 lung adenocarcinoma patient samples were collected. For each sample, the expression levels of *GSTP1* were determined by RT-qPCR and normalized to *TBP* as an internal control (early = 22, advanced = 21), ^**^p < 0.01. **(F)** Kaplan–Meier analysis of overall curves of patients with lung adenocarcinoma in high *GSTP1* expression (*n* = 221) and low *GSTP1* expression (*n* = 270). *P* < 0.05, log-rank test. **(G)** Hypothetical model illustrating cisplatin resistance mechanism in lung CSC cells. Lung CSC component is the cause of chemoresistance to cisplatin which is conferred by MEK/ERK transcriptional activation of *GSTP1*.

To verify whether the findings we made in LLC-SD mouse model of lung adenocarcinoma are applicable to lung CSC models derived from human adenocarcinoma, the human lung cancer cell line H1299 was used to generate H1299-SD cells (Figure 6Ai) using the same approach that we developed (36–38). Cisplatin resistance phenotypes observed in LLC-SD cells were similarly manifested in H1299-SD cells (Figures 6Aii,iii). *GSTP1* expression was also increased in H1299-SD compared with that in H1299 cells (Figure 6Aiv). More importantly, inhibition of *GSTP1* restored cisplatin sensitivity in H1299-SD cells *in vitro* (Figures 6Bi–iii). Further, the promoting effect of *GSTP1* on cisplatin resistance is regulated by MEK/ERK signaling (Figure 6C). This set of observations confirmed our main findings in the LLC-SD model and both collectively demonstrate that “lung CSC component is the cause of chemoresistance to cisplatin, which is conferred by MEK/ERK transcriptional activation of *GSTP1*.”

To assess the clinical relevance of *GSTP1* in lung adenocarcinoma, lung adenocarcinoma mRNA sequencing datasets from The Cancer Genome Atlas (TCGA) and ONCOMINE database were analyzed. The differential *GSTP1* expression between the cancer tissue and the normal lung was drastic and the elevation in lung adenocarcinoma tissues was profound (Figures 6Di,ii, Supplementary Figure 1B). Moreover, we collected 43 clinical paraffin samples of lung adenocarcinoma, including 22 cases of early adenocarcinoma (T1 and T2) and 21 cases of advanced adenocarcinoma (TIII and TIV). We analyzed *GSTP1* mRNA expression in this clinical cohort and found higher *GSTP1* expression in advanced lung adenocarcinoma compared to that in early stage lung adenocarcinoma (Figure 6E). Thus, *GSTP1* expression appears to correlate with disease staging. The low *GSTP1* expression in the non-cancerous lung tissue and staging-wise elevation could be explored for lung cancer diagnosis and merits further investigation.

We next analyzed the prognostic value of *GSTP1* expression by examining the relationship between *GSTP1* expression and lung adenocarcinoma progression using the publicly accessible TCGA (analyzed in Oncolnc website) and Kaplan-Meier Plotter (analyzed in Kaplan-Meier Plotter website) databases. High *GSTP1* mRNA levels are correlated with faster disease progression and a lower rate of survival in lung adenocarcinoma (Figure 6F, Supplementary Figure 1C). Thus, high *GSTP1* expression predicts poor prognosis.

Taken together, the bioinformatics analyses indicate that the findings we made using the mouse and human lung carcinoma CSC cellular models, regarding *GSTP1* mediation of cisplatin resistance, are of clinical relevance and importance.

## Discussion

Lung cancer management remains a challenge due to it being asymptomatic, having late presentation when it is metastatic and becoming resistant to anti-cancer therapies. Although platinum-based chemotherapy represents the standard first-line treatment for patients with advanced NSCLC, therapeutic outcome is compromised by the development of chemoresistance, relapse and disease recurrence (6, 7). Chemoresistance is a complex phenomenon, which may involve but is not limited to the reduction of drug accumulation, enhancement of DNA repair, impediment to apoptosis and alterations in cell cycle. Mechanistic research of the involvement of commonly studied multidrug resistant genes using human lung adenocarcinoma cell lines has yielded limited clinical results to help increase the understanding for overcoming resistance. While the role of lung CSCs in conferring multidrug resistance has been postulated, experimental evidence remains associative and lacks in depth mechanistic inquisition (12–14), partially due to the lack of stable cellular models of lung CSC.

The present study has made the following novel findings that contribute to an improved mechanistic understanding of clinically observed chemoresistance to platinum-based chemotherapies in advanced lung cancer:

First, lung CSCs are the source of chemoresistance. We developed and characterized the paired mouse Lewis lung adenocarcinoma cell line, LLC-Parental, and its CSC derivative cell line, LLC-SD (36–38), as well as paired human lung adenocarcinoma cell line, H1299-Parental, and its CSC derivative cell line, H1299-SD, that we generated using the same approach for this study (Figure 6) to overcome the obstacle of lacking stable lung CSC cellular models for mechanistic investigation. The sensitivity of both the LLC-Parental and H1299-Parental to chemotherapies, in deer contrast of the multidrug resistant phenotypes observed in the LLC-SD and H1299-SD CSCs, helped us to clearly identify the CSC component of both the mouse and human lung adenocarcinoma cell lines as the cellular component to confer multidrug resistance phenotype, in particular to cisplatin (Figures 1, 6). Thus, we have provided concrete experimental evidence demonstrating the involvement of lung CSCs in chemoresistance and have overcome the obstacle of the lack of stable cellular models of CSCs, including lung CSCs.

Second, expression of *Gstp1* is elevated in lung CSC cells, which can be further increased upon treatment with a panel of chemotherapy drugs. Using the two sets of cellular models for the screening of drug resistant genes that mediate multidrug resistant phenotype in the mouse and human lung CSC cells, we identified *Gstp1* among other candidates that was consistently expressed at higher levels in LLC-SD and H1299-SD cells and could be further increased upon treatment with a panel of chemotherapy drugs, in particular with cisplatin (Figures 1, 6).

Third, *Gstp1* confers cisplatin resistance in mouse and human lung CSC models, both *in vitro* and *in vivo*. Using lentiviral-mediated gene transfer, we generated LLC-SD (Figure 2) and H1299-SD (Figure 6) derivative cell lines, in which *Gstp1* expression was either inhibited or up-regulated. Through the bi-directional modulation of Gstp1 expression and *in vitro* (both LLC-SD and H1299-SD) (Figures 2, 6) and *in vivo* (LLC-SD) (Figure 2) characterization of the sensitivity to cisplatin, we can make a rather convincing conclusion that high *Gstp1* expression confers cisplatin resistance in experimental lung CSCs.

In the present study, we found elevated levels of *Gstp1* in response to multiple-drug treatment (Figure 1, Supplementary Figure 2), although *Gstp1* function is mostly studied under the context of platinum category of chemotherapeutic agents, due to its ability to remove the agents from the cells via the formation of Pt-glutathione. However, our findings are consistent with the literature reports that *Gstp1* expression could be regulated by transcriptional factors, including activating protein-1 (AP-1) and nuclear factor erythroid-2-related factor 2 (Nrf2), in response to multiple anticancer drugs, including chlorambucil, cyclophosphamide, cisplatin, DOX and mitoxantrone (41–43). Thus, Gstp1-mediated chemoresistance may not be limited to cisplatin and related anticancer agents, thus merits further studies.

Fourth, we have provided the first experimental evidence that transcriptional activation of *Gstp1* expression by MEK/ERK signaling underlies cisplatin resistance in lung CSC cells. Prior to our study, it was reported that in addition to MEK/ERK, both JNK/SAPK and p38 pathways were also affected by cisplatin treatment (44). In addition, Lin et al. reported that *Gstp1* expression can be upregulated by phosphorylation of Erk2 and by activating Nrf2 in response to methionine restriction (45). In the present study, we focused on examining MEK/ERK transcriptional regulation of *Gstp1* expression in response to cisplatin treatment in lung CSCs. Through lentiviral mediated siRNA interference of Mek1/2 expression, we confirmed the requirement of MEK/ERK activation to confer cisplatin resistance both *in vitro* and *in vivo* (Figure 3). We didn't stop here and took one step further which was to order the molecular relationship between activation of MEK/ERK signaling and transcriptional activation of *Gstp1* in mediating cisplatin chemoresistance. It was achieved by restoration of *Gstp1* expression in Mek1/2 silenced LLC-SD-shMek1/2 cells, which manifested concomitant *Gstp1* down-regulation, by overexpressing of *Gstp1* (Figures 5A,B). Restoration of *Gstp1* expression in LLC-SD-shMek1/2 cells reversed the cisplatin sensitive phenotype seen in LLC-SD-shMek1/2 cells to cisplatin resistance seen in shMek1/2+OEGstp1 cells, both *in vitro* and *in vivo* (Figure 5). These results confirm that transcriptional activation of *Gstp1* by MEK/ERK signaling pathway mediates cisplatin resistance in experimental lung CSCs.

Fifth, *GSTP1* expression is a poor diagnostic and prognostic marker for human lung adenocarcinoma, thus is of high clinical relevance. Prior to this study, the clinical relevance of *GSTP1* has not been well-explored. We conducted multi-scale analyses including the analysis of publicly available gene expression databases of human adenocarcinoma of lung, gene expression analysis of clinical paraffin blocks of lung adenocarcinoma as well as mouse and human lung adenocarcinoma CSC models. We found that *GSTP1* expression appears to correlate with disease staging (Figure 6). The low *GSTP1* expression in the non-cancerous lung tissue and staging-wise elevation could be explored for lung cancer diagnosis and merits further investigation. We also found that high *GSTP1* mRNA levels are correlated with faster disease progression and a lower rate of survival in lung adenocarcinoma (Figure 6F, Supplementary Figure 1C). Thus, high *GSTP1* expression predicts poor prognosis. Further, since the expression changes of *GSTP1* in response to chemo-therapeutic agents were specific to the lung CSCs, not the non-stem-like parental cells, *GSTP1* may also serve a lung CSC marker for evaluating the CSC content in clinical lung samples.

Taken together, the bioinformatics analyses indicate that the findings we made using the mouse and human lung carcinoma CSC cellular models, regarding *Gstp1* mediation of cisplatin resistance, are of clinical relevance and importance.

## Ethics Statement

This study was carried out in accordance with the recommendations of Institutional Animal Care and Use Committee of Chongqing medical University. The protocol was approved by Institutional Animal Care and Use Committee of Chongqing medical University. Experimental mice were raised in an accredited specific pathogen-free Animal Facility at Chongqing medical University. All protocols were approved by the Institutional Animal Care and Use Committee of Chongqing medical University and conducted with humane animal care. All of the formalin fixed paraffin embedded were obtained from department of pathology in the affiliated hospital of southwest medical university and received the approval of the institutional ethics committee of southwest medical university medical center. Moreover, this study obtained written informed consent from the participants.

## Author Contributions

JL and TY substantially contributed to conception and design, acquisition of data, analysis and interpretation of data, and drafting the article. YL, LK, ZS, and DL acquired part of the data. JW and HX contributed to conception and design, revising it critically for important intellectual content, and final approval of the version to be published.

### Conflict of Interest Statement

The authors declare that the research was conducted in the absence of any commercial or financial relationships that could be construed as a potential conflict of interest.

## Supplementary Material

Supplementary Figure 1 **(A)** GSEA analysis showed that *GSTP1* was positively associated with MEK/ERK signaling pathway in the TCGA lung cancer samples. **(B)** The Oncomine database mining analysis of *GSTP1* mRNA levels in Beer Lung, Stearman Lung, Hou Lung, and Okayama Lung grouped by lung cancer and normal specimens. **(C)** Kaplan-Meier analysis of overall survival (OS), progression-free survival (FS) and post-progression survival (PPS) curves of lung adenocarcinoma (LUAD) patients based on *GSTP1* mRNA expression (low vs. high).

Supplementary Figure 2 **(A,B)** The expression of resistance-related genes (*Gstp1, Abca2, Mrp1*) after 5-FU or paclitaxel treatment in LLC-SD cell lines by RT-qPCR, the tata-Box binding protein gene (*Tbp*) was reference gene. ^*^*p* < 0.05, ^**^*p* < 0.01.

Supplementary Figure 3 The expression of Gstp1 after cisplatin treatment in LLC-Parental and LLC-SD cells by western blotting, with GAPDH as a reference.

## Abbreviations

CSCs: cancer stem cells
LCSCs, lung cancer stem cells, NSCLC: non-small cell lung cancers
LUAD: lung adenocarcinoma.
