# Supplementary figures and images for: Transcriptional Activation of Gstp1 by MEK/ERK Signaling Confers Chemo-Resistance to Cisplatin in Lung Cancer Stem Cells
Source: Front Oncol. 2019 Jun 11;9:476. doi: 10.3389/fonc.2019.00476 (PMC6584806; doi:10.3389/fonc.2019.00476)

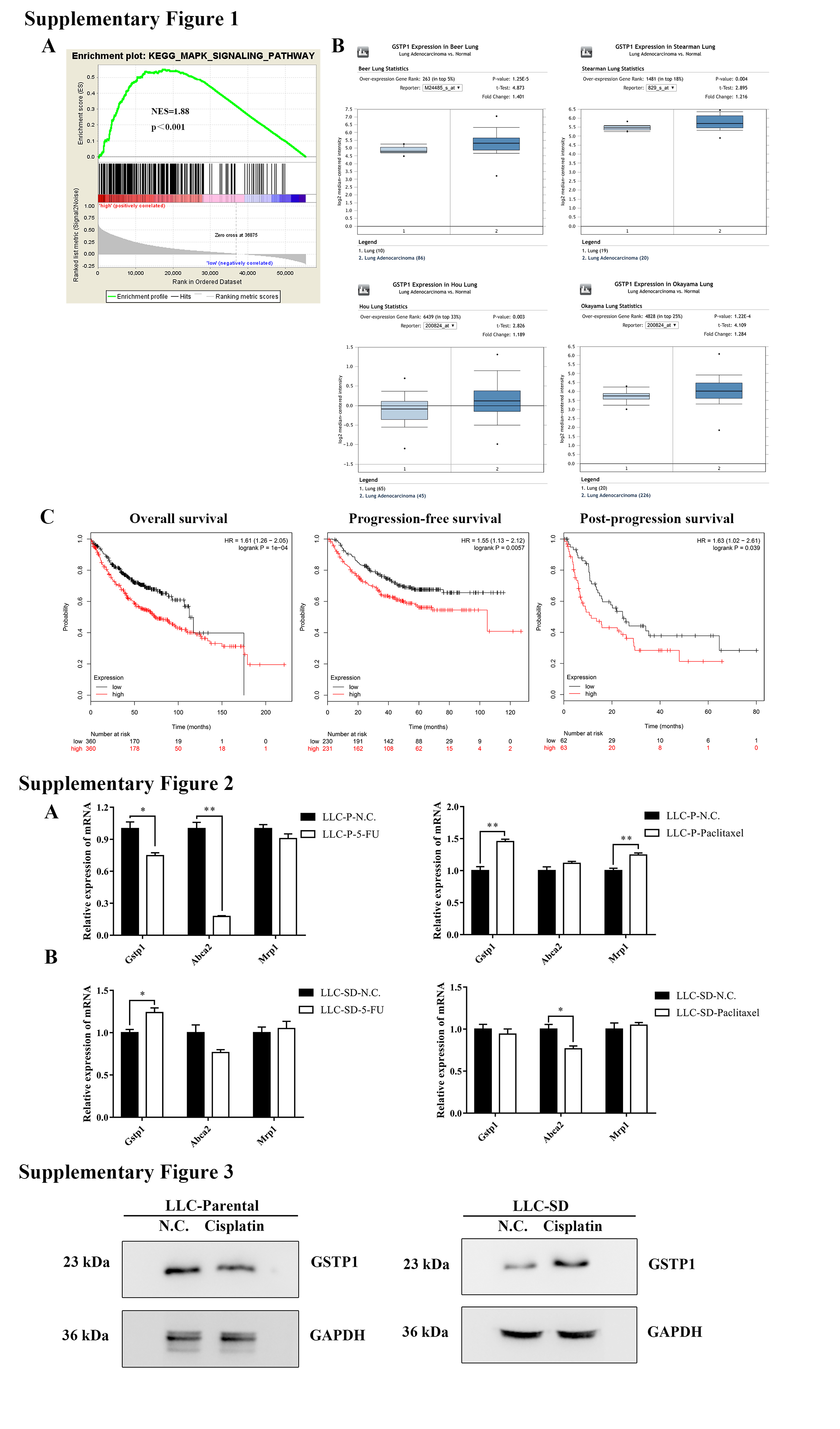

Supplement: Supplementary Figure 1 — (A) GSEA analysis showed that GSTP1 was positively associated with MEK/ERK signaling pathway in the TCGA lung cancer samples. (B) The Oncomine database mining analysis of GSTP1 mRNA levels in Beer Lung, Stearman Lung, Hou Lung, and Okayama Lung grouped by lung cancer and normal specimens. (C) Kaplan-Meier analysis of overall survival (OS), progression-free survival (FS) and post-progression survival (PPS) curves of lung adenocarcinoma (LUAD) patients based on GSTP1 mRNA expression (low vs. high). [file Image_1.JPEG]
